# Feasibility of a virtual reality-based approach to improve behavioral weight management outcomes

**DOI:** 10.1186/s40814-021-00865-5

**Published:** 2021-06-22

**Authors:** Suzanne Phelan, Sapna Peruvemba, David Levinson, Noah Stulberg, Aidan Lacy, Maria Legato, James P. Werner

**Affiliations:** 1 Center for Health Research, California Polytechnic State University, San Luis Obispo, CA USA; 2 Department of Kinesiology & Public Health, California Polytechnic State University, San Luis Obispo, CA USA; 3 Art & Design Department, California Polytechnic State University, San Luis Obispo, CA USA

**Keywords:** Virtual reality, Weight loss, Obesity, Lifestyle intervention, Behavioral treatment

## Abstract

**Background:**

Behavioral weight loss interventions promote clinically significant weight loss over 12 months, but weight regain remains problematic and a substantial proportion of participants do not achieve long-term weight loss maintenance. Novel methods are needed that instill habit strength for sustaining weight control behaviors long term. Virtual reality (VR) has the potential to provide opportunities within behavioral treatment for patients to practice desired weight control behaviors in the frequency and magnitude necessary to build durable habits. A pilot randomized trial was done to test the feasibility integrating virtual reality (VR) into standard behavioral weight loss treatment.

**Methods:**

Participants were 15 adults (43 years; 46.7% Hispanic), with overweight or obesity who were randomly assigned to a 4-week Standard Behavioral Weight Loss plus Non-Weight-Related VR app (i.e., Control Group) or Standard Behavioral Weight Loss plus Weight-Related VR app (i.e., Intervention Group). The Intervention’s VR tool was designed to enable practice of behavioral skills taught in weekly group meetings, including managing social and home environmental cues for eating and activity.

**Results:**

Participants were recruited over 3 months, and retention at the final assessment visit was high (86.6%). The VR footage and resulting app were rated as highly realistic (6.7 on a 10-point scale), and the VR program overall was rated as highly satisfactory (3.6 on a 4-point scale). Adverse effects of eye strain and motion sickness were minimal (~ 2 on a 7-point scale). As expected, the intervention and control groups both lost weight and unadjusted means (SD) averaged 3.4% (2.7) and 2.3% (3.6), respectively, over the 4 weeks. Overall, participants reported preferring a VR approach above traditional weight loss programs (rating of 5 on a 7-point scale).

**Conclusions:**

Future research is needed to develop and test the feasibility of using VR for other weight control skills with a larger sample size and longer evaluation period to determine if VR can improve standard behavioral weight loss outcomes by intensifying practice opportunities and building habit strength for weight loss maintenance.

**Trial registration:**

NCT04534088; date of registration: 09/01/2020, retrospectively registered.

**Supplementary Information:**

The online version contains supplementary material available at 10.1186/s40814-021-00865-5.

## Key messages regarding feasibility

- The feasibility integrating film-based virtual reality (VR) into standard behavioral weight loss treatment is unknown.
- Finding from this initial feasibility trial suggested that the VR film-based footage and resulting app were rated as realistic, acceptable, and preferred over traditional face-to-face weight loss intervention.
- Future research is needed to develop and test the feasibility of using VR for other weight control skills with a larger sample size and longer evaluation period to determine if VR can improve standard behavioral weight loss.

## Background

Behavioral weight loss interventions promote weight losses averaging 5–8% over 12 months [1]. This weight loss reduces odds of developing type 2 diabetes and improves cardiovascular disease risk factors [2, 3]. However, a substantial proportion of participants do not achieve long-term weight loss and clinically significant benefits [2, 3]. Novel methods are needed to build habit strength for practicing weight control behaviors and sustaining weight loss maintenance over the long term.

Virtual reality (VR) has potential to overcome limitations in standard behavioral weight loss by providing opportunities to practice desired weight control behaviors in the frequency and magnitude necessary for durable habit formation [4–8]. To date, most VR research in the field of obesity has involved participants experiencing VR during weekly sessions in a clinic or an inpatient setting [9], having them interact with a cartoon graphic, or use an avatar in a “virtual world” like a video game [10]. No known study has examined film-based, 360 video VR, which immerses a participant within the most realistic representation of space and narrative possible. By filming real-world environments, the 360 video VR has the potential to increase potency of the standard behavioral treatment approach, improve patient engagement and the ability to tolerate uncomfortable risk-related cues, and improve practice of desired behaviors in the frequency needed to promote habit strength in the behavioral skills of long-term weight loss maintenance.

The purpose of this pilot randomized trial was to develop a prototype and test the initial feasibility of integrating VR into standard behavioral weight loss treatment. We hypothesized that the study’s recruitment and retention approaches would be feasible and that the novel VR intervention would be acceptable. Exploratory hypotheses evaluated the intervention’s efficacy compared with the control condition in promoting 4-week weight loss.

## Methods

### Design

Virtually Healthy was a 4-week pilot randomized trial conducted at California Polytechnic State University, San Luis Obispo, CA, USA. Participants were randomly assigned to (1) Standard Behavioral Weight Loss plus Non-Weight-Related Virtual Reality (i.e., Control Group) or (2) Standard Behavioral Weight Loss plus Weight-Related Virtual Reality (i.e., Intervention Group). Participants completed assessments at study entry and after 4 weeks. The study was approved by the Institutional Review Board.

### Participants

Recruitment took place via flyers, social media, and presentations by investigators. Eligibility criteria included non-pregnant adults (age > 18 years), with overweight or obesity (BMI ≥ 25) who were able to speak and read in English. Participants had to be available on Thursday evenings when the group was held and have an iPhone 6 or higher for the VR app. Participants were excluded if they reported serious psychological problems or medical problems, a history of negative experiences associated with using VR, or moderate to severe motion sickness in vehicles. Participants who had never used VR were given the opportunity during a 10-min demonstration prior to enrollment. Those eligible were scheduled for a screening, consent visit, and randomization visit.

### Interventions

#### Both groups

Randomization to the Intervention or Control Group was computer-generated. Both groups received the same, 4-week behavioral weight loss program. The program was rooted in the Social Learning Theory (SCT) and prior behavioral interventions [2, 3, 11–19]. The weekly group sessions lasted 60 min and included 7–8 participants per group [20]. Trained lifestyle counselors led the group sessions. Each session started with a 15-min check-in on the progress in meeting weight, eating, monitoring, and activity goals and included presentation and discussion of a new lesson. The structured series of weekly lessons are shown in Table 1. Portable VR headsets for a phone were provided to use in VR homework. On weeks 2, 3, and 4, participants in both groups were asked to watch specific VR scenarios on three nights/week for 15 min before bed [21]. In the Control Group, these were non-weight, diet, and activity-related scenarios using the New York Times VR app (i.e., programs on marine life, nature, and sounds from around the world). For the Intervention Group, participants were asked to watch specific interactive VR scenarios via a provided app and headsets.

**Table 1:** The Virtually Healthy intervention program

Week 1	Weight loss and dietary goals, meal planning, and self-monitoring (no virtual reality homework)
Week 2	Calorie counting; assertiveness in restaurants (virtual reality homework)
Week 3	Stimulus control; modifying food cues (virtual reality homework)
Week 4	Exercise and modifying exercise cues (virtual reality homework)
Week 5	Program end and discussion of long-term weight loss maintenance (no virtual reality homework)

#### Intervention group

The intervention group’s VR scenarios complemented three weight control lessons: assertiveness in restaurants (session 2), modifying home eating cues (session 3), and modifying home exercise cues (session 4). The VR scenarios were scripted through an iterative process based on the input from the intervention staff, pilot participants, students, community members, and filmmakers until consolidation was reached. Actors were recruited and rehearsed and footage shot on an Omni GoPro rig, with six cameras and audio equipment. Singular, spherical video segments were stitched at 4K resolution using AutoPano Pro. Using the 3D software Unity, graphical elements were overlayed in the video and programmed for interactivity. The software was distributed through Apple iTunes Beta.

The VR restaurant scenario was ~ 10 min and placed the participant in a simulation where they were sitting at a circular restaurant table with co-workers who were ordering highly caloric and low-nutrient-dense appetizers, main courses, desserts, and drinks. At each ordering and eating/drinking segment, patients were given a virtual menu with several food/drinking options. The patient had to choose a healthy option (via gaze-based selection with timing indicators) in order for the video to proceed. If a participant selected a high-calorie option, a sound was heard and a red “X” appeared. After selecting a healthy option, a more positive sound was heard. Throughout the scenario, an interventionist was sitting at a nearby table periodically providing guidance and positive reinforcement.

The VR food cue scenario was created like a hide and seek game in the kitchen of a house. The interventionist again guided and encouraged the participant who was tasked with finding healthy cues in the kitchen, such as a bowl of apples, a food diary, meal plan, motivational poster, scale, meal replacements, and several other items. The simulation included entertaining distractors (e.g., child walking through the scene) and was designed to challenge patients to think about what household items could subtly encourage healthy food choices and ways they could modify their home to promote healthy eating.

The VR activity cue scenario was also created like a game and took place in the living room area of the house. Cues for identification included running shoes, motivational posters, pedometers, weights, an exercise bike, and other items. Intermittently, the interventionist would re-enter the scene, encouraging the patient and reinforcing selection of activity cues. The scenario included a dog and a person walking through as entertaining distractors. The scenario was designed to encourage the participant to identify the items that could prompt them to exercise more and to think about home environmental cues that shape behavior.

### Measures

Assessments were conducted at study entry and after 4 weeks. Participants received $10 for completing each assessment. In this initial study of the VR prototype, feasibility of recruitment was defined a priori and based on recruiting 15 participants over 3 months or less and resulting in a diverse sample (Hispanic > 20%). A refusal rate of < 10% and retention rate of > 80% were also expected. The feasibility of the intervention conditions was measured based on adherence to the intervention, defined as attending > 80% of intervention sessions and using the VR apps as prescribed (3 times/week). Acceptability and satisfaction were measured based on participants’ ratings of VR apps, the group facilitator, content, and overall impression at the month-long program. Additional questions assessed satisfaction specifically with the VR scenes developed for this study. The feasibility of the VR technology was also measured through an assessment of eye strain and motional sickness [22, 23]. Technical problems were also queried on a weekly basis and tracked by research assistants throughout the study. A demographics questionnaire at study entry assessed age, education, job status, race, and ethnicity. At study entry and after 4 weeks, weight was measured to the nearest 0.1 kg using a calibrated standard digital scale with participants in light clothing and no shoes. Standing height (mm) was measured in patients without shoes using a wall-mounted Harpenden stadiometer.

### Statistical analyses

Descriptive statistics, independent t tests, and chi-square tests were used to summarize information about recruitment, retention, process, and treatment acceptability measures for descriptive purposes. A general linear model was done to explore group differences in the 4-week weight loss both with and without adjusting the means for age and baseline body mass index (BMI).

## Results

**Fig. 1:**
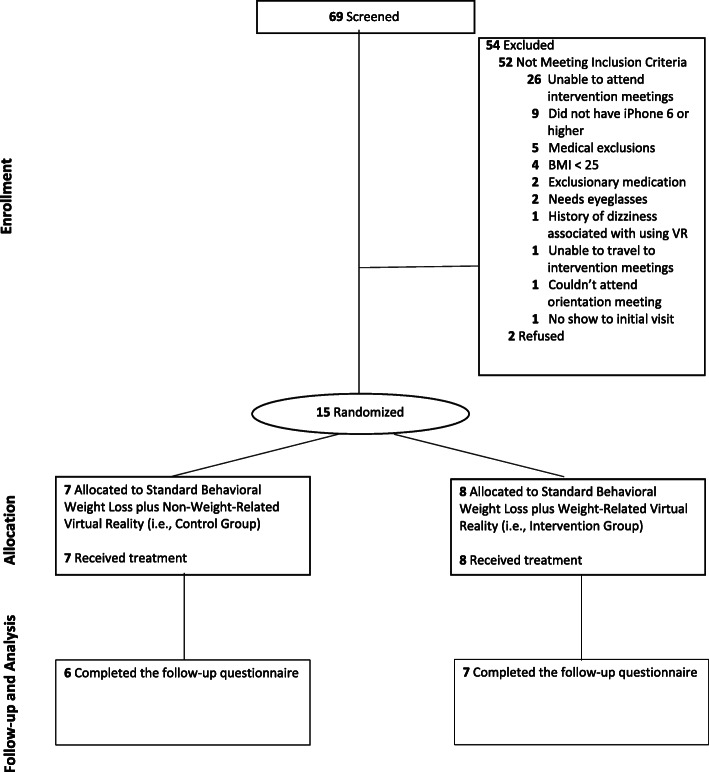
Flow chart

Recruitment was completed between December, 2018 and March, 2019, and the final assessment occurred in April, 2019. Retention at the final assessment was high with 85.7% (6/7) of controls and 87.5% (7/8) of intervention participants completing the visit; two participants did not complete the final assessment due to reported lack of availability on the final assessment day (n = 1) and unable to contact (n = 1). Figure 1 summarizes the participant flow and retention into the Virtually Healthy program. Of the 69 screened, 54 were ineligible; half of the ineligibles were due to unavailability to attend the intervention meeting time, which was restricted to one evening in this pilot study. Lack of an iPhone 6 was the next most common reason for ineligibility, affecting 16% (9/54) of those ineligible. Of the 69 screened, 2.9% (2/69) refused participation due to lack of interest in the program.

As shown in Table 2, 46.7% of participants were Hispanic. Participants were on average 43 years old, with a BMI in the obesity range. Most were female, married, or living with a significant other and reported an annual family income greater than $50,000/year. On average, 46.7% were college-educated. Half had some prior experience with VR and reported on average using VR 5 days/month. There were some between-group differences in sociodemographic factors, including BMI and age, which were adjusted for in analyses of weight change.

**Table 2:** Participant characteristics in Virtually Healthy. Participants were randomized to standard behavioral weight loss plus non-weight-related virtual reality (i.e., Control) or standard behavioral weight loss plus weight-related virtual reality (i.e., Intervention) groups.

	Overall (N = 15)	Control (N = 7)	Intervention (N = 8)
Age, mean (SD)	43.3 (14.7)	35.6 (14.8)	50.1 (11.3)
Female, %	86.7	38.5%	61.5%
Body mass index, mean (SD)	33.2 (5.3)	36.5 (4.9)	30.4 (4.0)
College degree, %	46.7	57.1%	37.5%
Hispanic, %	46.7	42.9%	50.0%
Family income > $50,000/year	80%	85.7%	75.0%
Employed for wages	53.3	71.4%	37.5%
Married or living with significant other	60.0	57.1%	62.5%
Wear glasses	40.0%	42.9%	37.5%
Prior experience with virtual reality	53.3%	71.4%	25%
Days per month currently use virtual reality, mean (SD)	4.9 (15.2)	2.3 (4.3)	0.3 (0.5)

Attendance at the 5 treatment sessions averaged 72% (3.6 [1.3] visits total) in the control and 88% (4.4 [1.1] visits) in the intervention group. Also, 54.7% (n = 4) of controls vs. 85.7% (N = 6) of intervention participants attended > 80% of visits. During the 3 weeks when VR homework was assigned, the app was used among controls and intervention participants on average 2.0 (1.4) and 2.0 (1.8) times per week, respectively, representing 66% of the amount prescribed (and including the data from individuals with technical problems, see below).

Satisfaction ratings for treatment components (meetings, assignments, goals) for both the control and intervention groups were high, with scores all above 3 on a 4-point scale where 1 = very dissatisfied and 4 = very satisfied (Table 3). Both groups also reported high satisfaction ratings and likelihood of recommending the program to others. Examining satisfaction with the virtually healthy app, ratings were also high (Table 4). Participants rated the scenarios on average as being realistic. Participants reported being overall satisfied with the VR homework and, in particular, with the restaurant scene (3.4 on a 4-point scale). The activity and food cue scenes received somewhat lower—but still satisfactory—ratings (2.6 and 2.4, respectively, on the same 4-point scale). For these scenes, difficulties in reading words on some objects (e.g., “food diary”) and objects being placed too far were reported by some participants. Participants reported preferring a VR approach above traditional weight loss programs (rating of 5 on a 7-point scale).

**Table 3:** Acceptability of the VR intervention and control programs

	Control N = 6	Intervention N = 7
Satisfaction ratings (scale where 1 = very dissatisfied and 4 = very satisfied )		
Weekly meetings with study interventionist	3.8 (0.4)	3.7 (0.5)
Weight, physical activity and diet goals	4.0 (0.0)	3.7 (0.4)
Weekly meetings with interventionist	4.0 (0.0)	3.8 (0.4)
Weekly behavioral assignments	3.7 (0.5)	3.6 (0.5)
How satisfied are you overall with the program you received from Virtually Healthy? (1 = very dissatisfied; 4 = very satisfied)	3.2 (1.2)	3.6 (0.8)
Would you recommend the program you received from Virtually Healthy to others? (1 = definitely not; 4 = definitely would)	3.3 (0.8)	3.7 (0.5)

*VR*, virtual reality; data presented as mean (standard deviation)

**Table 4:** Satisfaction with the intervention’s virtual reality scenarios

To what extent do you think that you would prefer a virtual reality over a more traditional weight loss approach? (1 = not at all prefer virtual reality; 7 = extremely prefer virtual reality)	4.7 (1.8)
Satisfaction (1 = very dissatisfied to 4 =very satisfied )	
VR homework in general	3.3 (0.5)
VR restaurant scene	3.4 (0.5)
VR activity cues	2.6 (1.3)
VR kitchen cues	2.4 (1.5)
How would you rate the distance of objects in the VR videos (1 = way too close; 5 = way too far)	3.3 (1.0)
How clear were the narration and instructions during the VR exercises? (1 = not clear at all; 10 = very clear)	7.3 (3.5)
Rate how difficult it was for you to find the objects in the Home Kitchen and Activity Cue simulations. (1 = too easy; 10 = too hard)	5.6 (3.2)
How would you rate your ability to look in the right place at the right time when people were talking in both scenarios? (1 = very easy; 10 = very hard)	5.3 (2.7)
Rate how realistic you felt the Kitchen scene portrayed in the VR video was to you. This question refers to the atmosphere of the scene, not the visual clarity of the video. (1 =not at all realistic; 10 = very realistic)	6.7 (2.4)
Rate how realistic you felt the Activity Cues scene portrayed in the video was to you. This question refers to the atmosphere of the space, not the visual clarity of the video. (1 =not at all realistic; 10 = very realistic)	6.7 (1.9)
Rate how effective you found the selection menu in the restaurant scenario (1 = not effective; 10 = very effective)	5.6 (2.2)
To what extent were your eyes strained during the VR experiences? (1 = not at all; 7 = extremely)	2.6 (1.8)
To what extent did you experience motion sickness or nausea during the VR experiences? (1 = not at all; 7 = extremely)	2.1 (1.9)

*VR* virtual reality; data presented as mean (standard deviation)

Little-to-no eye strain or feelings of motion sickness were reported (Table 4). Two technical problems were reported in the intervention group. One person reported that they were unable to download the app due to download permissions that restricted access on the phone. Another person reported receiving a message that the app had expired during the last week of the intervention group, which was unresolved prior to project completion.

Both the control and intervention groups lost weight over the 4-week program with unadjusted means (SD) averaging 2.7 (4.4) vs 2.6 (2.1) kg, respectively, and adjusted means (SE) that accounted for baseline BMI and age averaging 2.9 (1.7) and 2.4 (1.5) kg, respectively. As a percentage of initial body weight, the Control Group vs. Intervention Group lost an average (SD) unadjusted mean of 2.3% (3.6) vs. 3.4% (2.7), respectively over the 4 weeks; in adjusted analyses, these means (SE) were 3.2% (1.5) and 2.7% (1.4), respectively. Examining the proportion of participants who lost 5% or more of initial body weight, 16% (1/6) of participants in the Control Group compared with 43% (3/7) of participants in the Intervention Group met this criterion.

## Discussion

To our knowledge, this is the first development and usage of VR film as a tool to improve standard behavioral treatment for weight management. Preliminary evaluation of the program over 4 weeks suggested that the integration of VR into standard treatment was feasible and acceptable. Recruitment was conducted in a timely fashion, and refusal rates were low. The recruited sample represented a diverse patient population, including nearly 50% of participants who self-identified as Hispanic and an equal proportion (53%) who had never used VR previously. The VR footage and resulting app were rated as realistic with few-to-no adverse effects. Both groups produced weight losses that were in line with expectations. Future research is now needed to develop and test the feasibility of this approach using more VR scenarios, a larger sample size, and a longer evaluation period.

The VR scenarios complemented the weight control strategies discussed during weekly standard behavioral treatment meetings. The presence of the virtual interventionist (played by an actress) who provided guidance and positive reinforcement appeared particularly effective. One problem with the cue-finder games was that some of the home environment cues that contained writing (e.g., motivational poster, food diary) were placed too far away for easy reading by some participants. Future shoots should ensure optimal lighting and large font for naming food diaries or other positive cues that contain words. Only a few technical issues were reported. With these caveats, participants reported on average that they would prefer the VR approach over standard behavioral programs.

Participants in the current study were instructed to watch the videos three times per week. Investigators initially considered a prescription to use the VR more frequently but decided against the idea in order to prevent potential boredom. However, investigators’ initial concerns of boredom were not validated; satisfaction ratings were high. Although both groups used the VR app less than prescribed (on average two times per week vs. 3 days prescribed), this appeared to reflect reduced adherence due to a few weeks with technical problems and one participant using her partner’s phone. These could be addressed in future research that included more training in installing and using the VR apps and providing technology to those in need. Given the importance of repetition in habit formation [24–26], future implementation of this approach should consider prescribing daily or more frequent repetition of the VR assignments. Future research is also needed to determine whether VR integration into standard behavioral treatment offers more frequent opportunities for patients to engage in, practice, and develop habit strength for long-term weight control behaviors.

Other weight control behaviors that could be practiced through VR include selecting portion sizes and navigating selection of foods in grocery stores, buffets, and other social gatherings, and choosing active over non-active activities. VR could facilitate frequent practice of non-eating responses to highly palatable foods, such as salty snacks, cookies, candy, and sugary drinks [27] and in response to strong emotional cues, such as boredom or cravings. Although visual cues (e.g., pictures and videos) appear most effective [28], additional sensory stimuli could be considered to make the experience “4D”. Participants could be instructed to practice the VR homework during high-risk times for eating in the absence of hunger, such as during the evening or during other sensory states (e.g., after an alcoholic beverage) [29]. Future research could include biometric measures of emotional reactivity and lab-based measure of eating to provide objective means of evaluating the efficacy of this approach.

Few prior studies have examined VR approaches to weight loss. The most relevant work in this area has been led by investigators in Italy who have developed and refined a VR computer-generated imagery animation environment to assist during clinical sessions for treatment of body image disturbances in patients with severe obesity and binge eating [30–33]. The VR program was adapted for use in a cognitive behavioral treatment (CBT) for patients with obesity in an inpatient setting. VR + CBT appeared to produce greater 6-month weight loss than CBT alone (13 kg loss vs. 6–9-kg loss) [30] and facilitate weight loss maintenance through 1 year in completer [34] but not intent-to-treat analyses [35]. However, the comparison groups in these studies were unmatched in contact time (i.e., the VR group received an additional 15 sessions), and the CBT weight loss approaches tested to “unlock negative memory of the body” [34] were somewhat unconventional. Importantly, these graphics-based VR approaches have the patient select an avatar in a “virtual world” and interact with settings like a video game [10]. Graphical scenes have the advantage of being edited quickly and potentially more rapidly and tailored an individual patient’s triggers. However, the current study’s use of film, while potentially less flexible, has the great advantage of being realistic, as suggested in the current study. Experimental designs that control for contact time and include populations with overweight and obesity who are outside of the inpatient setting are needed to advance this nascent area of research.

This current study has strengths and some weaknesses. A prototype of a film-based VR method was developed and tested. Feasibility was examined, and the study included a randomized trial methodology. However, the study was limited by small sample size and short-term evaluation. Key measures, such as habit strength, coping, and changes in specific behaviors were not evaluated. It is possible that feasibility and satisfaction differed by gender, BMI, and age, but these too were not analyzed given the small sample size. Finally, the interventionists were not masked to treatment assignment, which could have biased intervention delivery and should be addressed in future research.

## Conclusions

In conclusion, the goal of this small pilot study was to establish initial feasibility of a film-based VR habit formation tool. Future research is needed to determine whether and how this approach can improve long-term weight loss maintenance [36]. VR has the potential to improve upon standard behavioral weight loss treatment by offering more frequent opportunities for patients to practice behavioral modification skills, including coping with uncomfortable situational cues and drives to eat, and by providing more positive reinforcement and encouragement from an interventionist in life-like situations [37, 38].

## Supplementary Information

**Additional file 1.** CONSORT 2010 checklist of information to include when reporting a pilot or feasibility trial*.

## Data Availability

A de-identified version of the dataset will be made available upon reasonable request.

## Abbreviations

VR: Virtual reality
BMI: Body mass index
Kg: Kilograms
